# High Inter-Individual Diversity of Point Mutations, Insertions, and Deletions in Human Influenza Virus Nucleoprotein-Specific Memory B Cells

**DOI:** 10.1371/journal.pone.0128684

**Published:** 2015-06-18

**Authors:** Sven Reiche, Yamen Dwai, Bianca M. Bussmann, Susanne Horn, Michael Sieg, Christian Jassoy

**Affiliations:** 1 Institute of Virology, Faculty of Medicine, University of Leipzig, Leipzig, Germany; 2 Department of Biology, Faculty of Natural Sciences, Tishreen University, Latakia, Syria; Icahn School of Medicine at Mount Sinai, UNITED STATES

## Abstract

The diversity of virus-specific antibodies and of B cells among different individuals is unknown. Using single-cell cloning of antibody genes, we generated recombinant human monoclonal antibodies from influenza nucleoprotein-specific memory B cells in four adult humans with and without preceding influenza vaccination. We examined the diversity of the antibody repertoires and found that NP-specific B cells used numerous immunoglobulin genes. The heavy chains (HCs) originated from 26 and the kappa light chains (LCs) from 19 different germ line genes. Matching HC and LC chains gave rise to 43 genetically distinct antibodies that bound influenza NP. The median lengths of the CDR3 of the HC, kappa and lambda LC were 14, 9 and 11 amino acids, respectively. We identified changes at 13.6% of the amino acid positions in the V gene of the antibody heavy chain, at 8.4 % in the kappa and at 10.6 % in the lambda V gene. We identified somatic insertions or deletions in 8.1% of the variable genes. We also found several small groups of clonal relatives that were highly diversified. Our findings demonstrate broadly diverse memory B cell repertoires for the influenza nucleoprotein. We found extensive variation within individuals with a high number of point mutations, insertions, and deletions, and extensive clonal diversification. Thus, structurally conserved proteins can elicit broadly diverse and highly mutated B-cell responses.

## Introduction

The repertoire of antigen-specific B cells in humans remains largely unexplored due to difficulties in generating large sets of antibodies with defined specificity. The main obstacle to the generation of antigen-specific antibodies has been the isolation and selection of the cells [1]. In recent years, technical advances have been made in generating antigen-specific human monoclonal antibodies. One important advance has been the production of recombinant antibodies through the amplification and cloning of B cell receptor (BcR)/antibody genes from single B cells [2, 3]. Two of the earliest studies using the BcR amplification technique generated influenza-specific antibodies from plasmablasts and HIV gp120-specific and gp41-specific antibodies from memory B cells [4, 5]. BcR amplification generates greater numbers of antibodies compared with other methods, which include laborious transformations of cells with Epstein-Barr virus and the generation of hybridomas, providing new opportunities to gain insight into the compositions of antigen-specific B cell repertoires.

Single-cell antibody cloning has been used to generate and characterize antibodies against influenza virus [5–7], HIV [4, 8, 9], rotavirus [10], and *Plasmodium falciparum* [11]. *P*. *falciparum*-specific antibodies derived from atypical memory B cells had more point mutations than those from classical memory B cells [11]. Rotavirus-specific B cells showed an overrepresentation of the heavy chain (HC) gene family IGVH4 [10]. Scheid et al. obtained monoclonal antibodies by isolating memory B cells from individuals with chronic HIV infection [4]. Between 5 and 51 distinct B cell variants were identified within each individual, indicating that the BcR repertoires of the HIV glycoprotein-specific memory B cells are highly diverse. HIV gp140-specific B cells had increased numbers of point mutations and more frequently used the HC V-gene family IGVH1, the κ versus the λ light chain (LC) gene family, and the J-gene variants Jκ2 and Jκ5 compared with nonspecific B cells from the same individuals [12]. The fine characterization of broadly neutralizing HIV-specific antibodies revealed exceptionally high mutation rates, long CDR3s, and a high percentage of insertions and deletions [13–15]. Wrammert et al. generated 61 influenza-specific monoclonal antibodies from the plasmablasts of five human donors after boost vaccination with influenza vaccine. Most of the antibodies were specific to the influenza virus hemagglutinin glycoprotein. Genetic analyses of plasmablasts enriched for influenza-specific B cells from a larger number of donors showed extensive accumulation of somatic mutations: Influenza-specific plasmablasts showed increased numbers of point mutations compared with nonspecific-memory and germinal-center B cells. The influenza-specific B cell repertoires were highly restricted and were dominated in some donors by only a few plasmablast clones, indicating expansions of a small number of B cells [5].

Humans are typically infected with influenza during early childhood. At four years of age, the prevalence of antibodies against influenza A and B viruses is 80% [16, 17]. Reinfections occur repeatedly during the average lifetime. Reinfections are facilitated by the high mutation rate of viral replication and the selection of viruses with escape mutations in the hemagglutinin protein, which is the primary target of neutralizing antibodies. We previously observed that virtually all adults, including healthy individuals and individuals infected with HIV, have memory B cell responses against the influenza nucleoprotein (NP) [18–20]. Compared with that of the hemagglutinin protein, the evolutionary rate of the viral NP, which is not a target for neutralizing antibodies, is three to four times lower, indicating that the amino acid sequences of the NP among different viral isolates are better preserved than those of the hemagglutinin protein [21].

In this study, we examined the diversity of influenza NP-specific memory B cells to characterize the memory B cell response to a well-conserved influenza protein. We obtained blood samples from healthy individuals prior to and following influenza vaccination. We isolated influenza NP-specific memory B cells in a highly efficient way and characterized the BcRs of the cells. We found that influenza NP-specific antibodies could be obtained without prior influenza vaccination. Our results indicate that the influenza NP-specific memory B cell response in humans is broad and diverse and is unique within each individual.

## Materials and Methods

### Study subjects

We obtained whole-blood samples from four healthy adult volunteers (designated D1–D4). The volunteers included two males and two females born in 1987 (D1, D2), 1981 (D3) and 1961 (D4). Individual D1 had no history of influenza vaccination; the blood from that individual was obtained at two time points (during June and October) outside of the influenza season. The blood from individual D2 was obtained prior to and two weeks after an influenza boost vaccination with a split-virus vaccine (Mutagrip, Sanofi Pasteur). The blood from individual D3 was obtained two weeks after an influenza boost vaccination with a split-virus vaccine (Afluria, CSL Biotherapies). The blood from individual D4 was obtained two weeks after an influenza boost vaccination with a hemagglutinin/neuraminidase subunit vaccine (Grippeimpfstoff ratiopharm 2011/2012, Abbott Biologicals B. V.). The vaccines were based on the influenza strains A/California/07/2009 (H1N1), A/Perth/16/2009 (H3N2) and B/Brisbane/60/2008 used for vaccination in 2011/2012 in the Northern hemisphere.

### Ethics statement

Participants provided written informed consent to participate in the study. The Ethics Committee of the Medical Faculty of the University of Leipzig approved the study including the informed consent procedure.

### Isolation of antigen-specific memory B cells

Peripheral blood mononuclear cells (PBMCs) were prepared from heparinized whole-blood samples (120–150 ml) by density-gradient centrifugation using a ficoll cushion. B cells were isolated from the samples using immunomagnetic anti-CD19 beads (Dynal Pan CD19 kit, Life Technologies, Inc.). IgM^+^ cells were removed from the samples using anti-human IgM microbeads (Miltenyi Biotec). Influenza (H3N2) NP-specific cells were isolated from the IgM^-^ B cell fractions with streptavidin-coated superparamagnetic beads (CELLection Biotin Binder Kit, Life Technologies, Inc.) coated with biotinylated recombinant influenza NP from influenza virus strain A/HongKong/68 (H3N2) [22]. To induce plasma B cell differentiation, the isolated IgM^-^ cells were cultured in RPMI-1640 medium with 10% fetal calf serum (FCS) and antibiotics at 37°C in round-bottomed 96-well plates in the presence of a mixture of mitogenic agents including pokeweed mitogen (10 ng/ml, PWM, Sigma-Aldrich, Inc.), *Staphylococcus aureus* lysate (equivalent IgG binding capacity of 0.12 μg/ml, Sigma-Aldrich, Inc.), interleukin (IL)-2 (100–200 ng/ml, Proleukin, Novartis AG), IL-10 (0.025 μg/ml, Hiss Diagnostics GmbH), and phosphorothioated CpG ODN-2006 (1 μg/ml, Metabion GmbH) [18]. The cultured cells were counted after 6 days of growth. One or two cells from each culture were resuspended in PBS in 0.2-ml PCR tubes and frozen at -20°C until further analysis. As a control, activated B cells depleted of IgM^+^ and influenza NP-specific B cells from individuals D3 and D4 were aliquoted and frozen in the same fashion.

### ELISpot test

A fraction of the cells was used to determine the purity of the antigen-specific isolation. For that test, equal numbers of B cells were plated in two wells of an ELISpot plate (Milllipore, Inc.) coated with recombinant influenza NP (1 μg/well) or goat anti-human IgG (F(ab’)_2_) (1 μg/well; Dianova GmbH). After 20 h at 37°C, the plates were washed, alkaline phosphatase-conjugated goat anti-human IgG (Dianova GmbH) was added, and the cells were incubated for 2 h at 37°C. The plates were developed using the AP Conjugate Substrate Kit (Bio-Rad Laboratories, Inc.). Spots were counted using the AID ELISpot 04 plate reader (Autoimmune Diagnostika GmbH). The purity of the isolation was determined by calculating the ratio of antigen-specific cells to IgG-secreting cells.

### Antibody expression plasmids

The expression plasmids for the HC and LC are based on the plasmid pVITRO2-mcs (Invivogen, www.invivogen.com). For the HC, an expression cassette containing the leader sequence of the *Homo sapiens* Ig heavy constant gamma 1 gene (G1m marker, NCBI reference: BC073782), the variable region and the constant region of IgG1 were inserted between restriction sites AgeI and AvrII. Restrictions sites ClaI and SalI were introduced at the end of the leader sequence and immediately after the J gene region, respectively. The ClaI/SalI fragment that spans the variable region was replaced by a non-Ig sequence of 4601 base pairs as a placeholder. For the κ and λ LCs, an expression cassette containing the leader sequence of the *Homo sapiens* Ig kappa light chain (T6J/k, NCBI reference: AF027158), the variable region and the constant region of the κ LC was inserted between the restriction sites AgeI and SalI. A BsiWI restriction site was introduced at the end of the leader sequence and a XhoI site was generated downstream of the J gene in the constant region. The variable region fragment was subsequently replaced by a non-Ig sequence of 2817 basepairs.

### Amplification and gene cloning

RNA from the frozen single cells was reverse transcribed at 50°C for 60 min in a 20 μl reaction mixture containing 1 μl oligo dT18 primer (10 mM), 1 μl dNTP-Mix (10 mM each nucleotide), 1 μl 0.1 M DTT, 0.5 μl RnaseOUT (Life Technologies, Inc.), and 100 U Superscript III (Life Technologies, Inc.). The reaction was inactivated at 70°C for 15 min. IGH transcripts were amplified using 2.5 μl cDNA. IGκ and IGλ transcripts were amplified using 5 μl cDNA. PCR amplification was performed using derivatives of previously published primer sequences, which were modified to contain restriction-enzyme cleavage sites suitable for our HC and LC cloning plasmids described above [2, 3]. The first PCR was performed with 0.5 μl 5’ and 3’ primer mix (10 pM each primer), 1 μl MgCl_2_ (25 mM), 0.5 μl dNTPs (10 μM, Life Technologies, Inc.), and 1.25 U Hotstar Taq DNA polymerase (Qiagen). After an initial activation step at 95°C for 15 min, the thermocycler performed 40 cycles of 95°C for 30 s, 55°C for 30 s, and 70°C for 30 s. The second PCR was performed with 2.5 μl (IgH) or 5 μl (Igκ or Igλ) product from the first PCR, 0.75 μl 5’ and 3’ primer mix (10 pM each primer), 1 μl MgSO_4_ (25 mM), 2.5 μl dNTPs (2 mM each, Merck Millipore), 1 μl DMSO, and 5 U KOD hotstart DNA polymerase (Merck Millipore). After 40 cycles at 95°C for 20 s, 65°C for 10 s, and 72°C for 10 s, the PCR products were purified by agarose gel electrophoresis and DNA extraction (Qiaquick, Qiagen), digested with restriction enzymes. The amplified HCs were cloned between the ClaI and SalI restriction sites and the LCs between BsiWI and XhoI in the HC or LC expression plasmids.

### Recombinant antibody production

Antibodies were produced using human embryonic kidney 293T fibroblasts. Five times 10^6^ cells per 6-well plate were cultured in DMEM (Life Technologies) supplemented with 10% FCS (Biochrom AG) for 16 h. Cells in each well were transfected with 5 μg of the HC and LC plasmid using calcium phosphate precipitation. After 24 h, the medium was replaced with fresh DMEM plus 10% FCS. After 48 h, the supernatants were collected and frozen until further use.

### ELISA

Influenza NP-specific reactivity was tested by ELISA. Ninety-six-well microtiter plates (Greiner Bio-one GmbH) were coated overnight with recombinant influenza NP linked to an MBP fusion protein (0.1 μg/well) or MBP alone (0.05 μg/well) in coating buffer (0.2 M NaHCO_3,_ pH 9.6). The plates were washed with phosphate-buffered saline/0.05% Tween-20 (PBST) and blocked with 3% bovine serum albumin in PBST at 37°C for 1.5 h. Cell-culture supernatants were diluted 1:10 in blocking buffer and incubated for 2 h at 37°C. The plates were washed, horseradish peroxidase-conjugated rabbit anti-human IgG (Dako GmbH) was added, and the plates were incubated at 37°C for 1.5 h. The plates were washed again and incubated for 30 min at room temperature with tetramethyl benzidine (TMB) solution. The reaction was stopped with H_2_SO_4_, and the optical density (OD) at 450 nm was determined. OD values obtained with the MBP control protein were subtracted. The resulting OD values of antibody supernatants were regarded as positive if they exceeded 0.3.

### Establishing a filter for mutational artifacts

The PCR polymerases used for amplification and Sanger sequencing are prone to errors, and PCR-amplified sequences may contain nucleotide mutations as *in vitro* artifacts. On the other hand, boost vaccination can cause the rapid diversification of clonal lineages [5]. To differentiate between PCR artifacts and clonal diversity, we calculated the PCR error rate. The calculation was based on the observation that the frequency of memory B cells specific to a single viral protein is rarely above 0.1% [18, 20, 23]. That makes it unlikely to find genetically identical cells within, for example, a sample of 100 nonselected memory B cells. To determine the PCR artifact frequency, B cells depleted of influenza NP-specific cells were activated *in vitro* in the same way that the antigen-specific B cells were activated. BcR gene cloning and analysis showed duplicates and several small groups of cells with identical V, D, and J genes, indicating that the cells had multiplied *in vitro*. Taking the sequence that was closest to the germ-line V sequence in each group as a reference, the error rate was calculated as the number of nucleotide deviations among the sequences of the other group members. Approximately 26% of the nucleotide sequences differed from the corresponding reference sequences. The overall frequency of point mutations was 0.15%. The individual antibody sequences differed by up to four nucleotides, corresponding to one or two amino acids. Based on those findings, we used two amino acids as a filter to differentiate between potential *in vitro* artifacts and differences induced *in vivo*. The sequences of influenza NP-specific cells that showed three or more amino acid changes were regarded as HC or LC sequences from unique B cells.

### Data analysis and statistics

The raw sequence data were trimmed to the relevant lengths, analyzed for open reading frames, and aligned using Geneious R6 DNA analysis software (Biomatters Ltd, Auckland, New Zealand). The B cell receptor HC and LC gene usage, the CDR3 length, and the numbers of somatic amino acid changes and somatic insertions and deletions were determined according to the IMGT system using the IMGT/V-QUEST and IMGT/High V-QUEST software [24, 25]. The data were further analyzed and plotted using Microsoft Excel and R [26]. Two-sided Wilcoxon rank tests were used to determine the statistical significance of the differences between sets of data from independent samples.

## Results

### Effective purification of influenza NP-specific memory B cells with immunomagnetic beads

**Fig 1:**
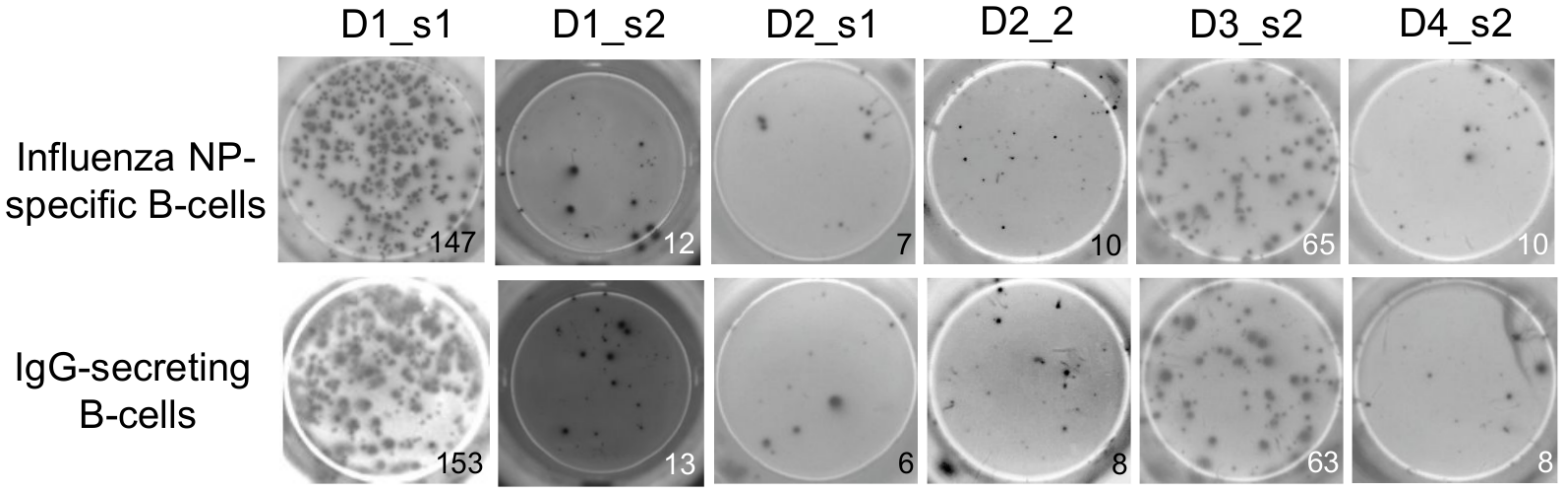
Purity of influenza virus NP-specific B cells. Influenza NP-specific B cells were isolated with immunomagnetic beads, cultured, split and tested by ELISpot for antibodies specific to the influenza virus NP (upper row) and for the secretion of IgG (lower row). Two samples were tested from individuals D1 and D2, respectively, and one sample was tested from individuals D3 and D4, respectively. Each spot in the wells of the ELISpot plate indicates a single antibody-secreting B cell. The quantity of antibody-secreting cells is indicated by the number below the well.

Isolation of influenza virus NP-specific memory B-cells consisted of several steps of cell separation. Influenza NP-specific B-cells were isolated from CD19+ B-cells depleted of IgM+ cells using antigen-coated superparamagnetic beads. The recombinant influenza virus NP was bound to streptavidin-pre-coated beads via a biotinylated signal sequence. Cells that bound to the beads via the antigen receptor were stimulated *in vitro*. A minimum of 200–500 cells was required so that the cells survived several days in culture indicating that the B-cells provided an autocrine or paracrine survival signal. After stimulation, a fraction of the cells was used to measure antibody secretion and to determine the percentage of influenza NP-specific B-cells. The ELISpot results revealed almost identical numbers of spots in pairs of wells containing IgG-secreting cells and influenza NP antibody-secreting cells, respectively. Hence, virtually all of the IgG-producing cells were influenza NP-specific (Fig 1).

### Relatedness of antibody cloning and gene amplification efficiency

**Fig 2:**
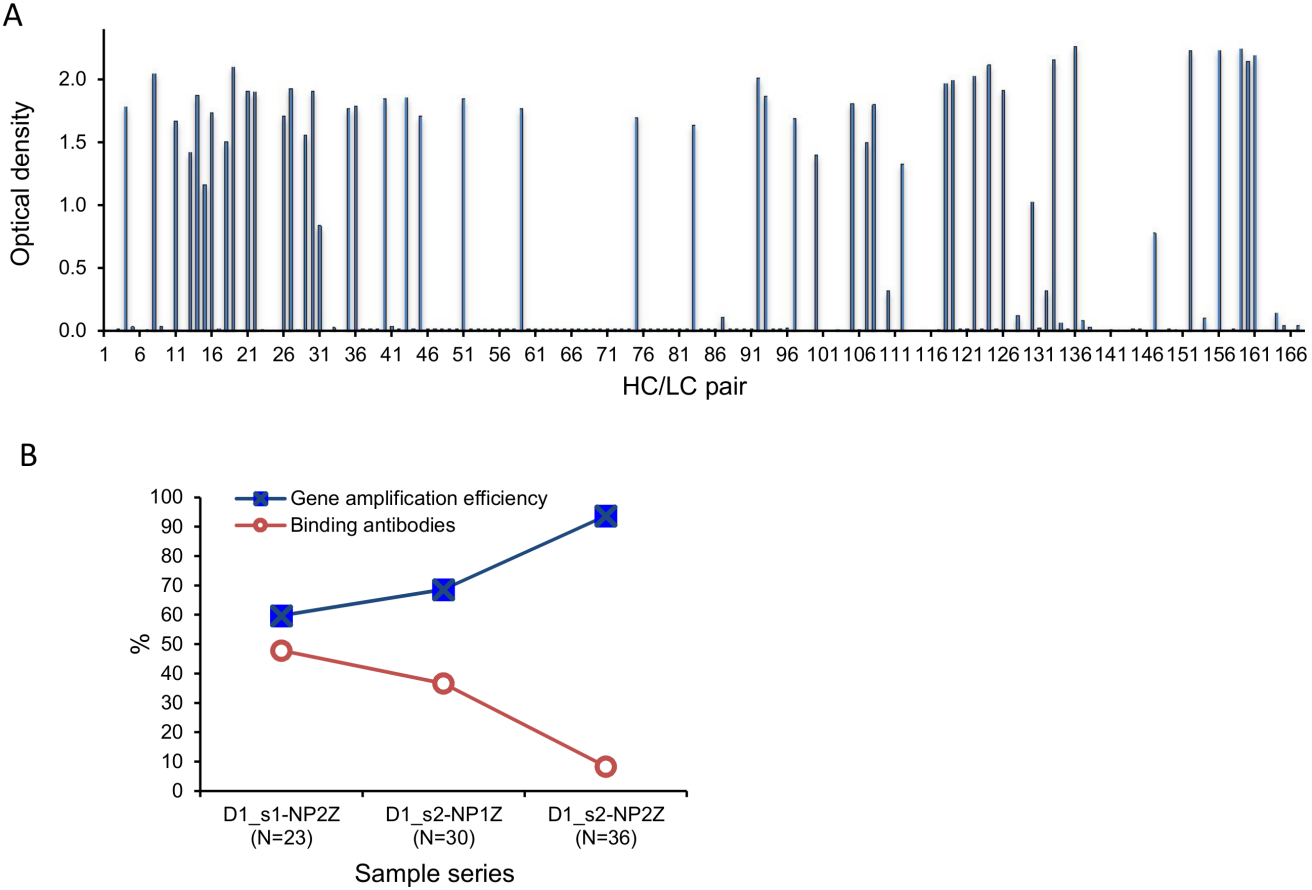
Influenza NP binding and gene amplification efficiency. **A**) Optical density values obtained in the influenza NP ELISA with the supernatants of cells transfected with pairs of HC and LC sequences from the same B cell vial. **B**) Association of gene amplification efficiency and the percentage of vials from which influenza NP-binding antibodies were obtained. Three sets of samples from individual D1 from two time points were analyzed. N is the number of tubes that gave rise to HC/LC sequence pairs. The gene-amplification efficiency varied (squares). The fraction of HC/LC pairs that yielded influenza NP-binding antibodies (circles) decreased with increasing gene amplification frequency.

In total, we amplified 180 HC, 177 LCκ and 54 LCλ sequences with intact reading frames. For most HCs, one or, occasionally, two LCs were obtained from the same sample, leading to 167 HC/LC sequence pairs. Forty-seven HC/LC plasmid pairs (28.1%) gave rise to influenza NP-specific antibodies (Fig 2A). The gene amplification efficiency in the different sets of B cell samples was variable and ranged from 59 to 93%. The percentage of HC/LC pairs that gave rise to influenza virus NP-specific antibodies was negatively correlated with the percentage of isolated B cell samples from which gene sequence pairs could be amplified. Thus, a high yield of HC and LC sequences from a given sample set resulted in a low number of functional antibodies from that sample and vice versa. At an amplification efficiency of 59.7% (sample set D1_s1-NP2Z), 48% of the HC and LC yielded NP-binding antibodies (Fig 2B). We conclude from this observation that the lower than expected functional antibody yield was primarily due to mismatching heavy and light chains amplified from different B cells in vials with more than one cell.

### Usage of multiple germ-line V genes

To measure the diversity of the BcRs, we determined the germ-line origin of the V, D, and J HC genes and that of the LC V and J genes using the IMGT HighV-QUEST software [24, 25]. After applying the artifact filter, 123 unique HC sequences, 112 unique LCκ sequences and 38 unique LCλ sequences were obtained for further analysis. In each of the donor individuals, we found between 19 and 51 unique HC sequences, 19 and 44 unique LCκ sequences, and 4 and 18 unique LCλ sequences. Between five and 22 HC and LC chains matched in each donor individual, giving rise to 43 genetically distinct antibodies that bound influenza NP (Table 1).

**Table 1:** Number of unique influenza NP-specific BcR sequences and genetically distinct influenza virus NP-binding antibodies from each subject.

	Unique sequences			Influenza virus NP-
Subject	HC	LCκ	LCλ	binding antibodies
**D1**	51	44	18	22
**D2**	29	24	8	7
**D3**	19	19	8	9
**D4**	24	25	4	5
**Total**	**123**	**112**	**38**	**43**

**Fig 3:**
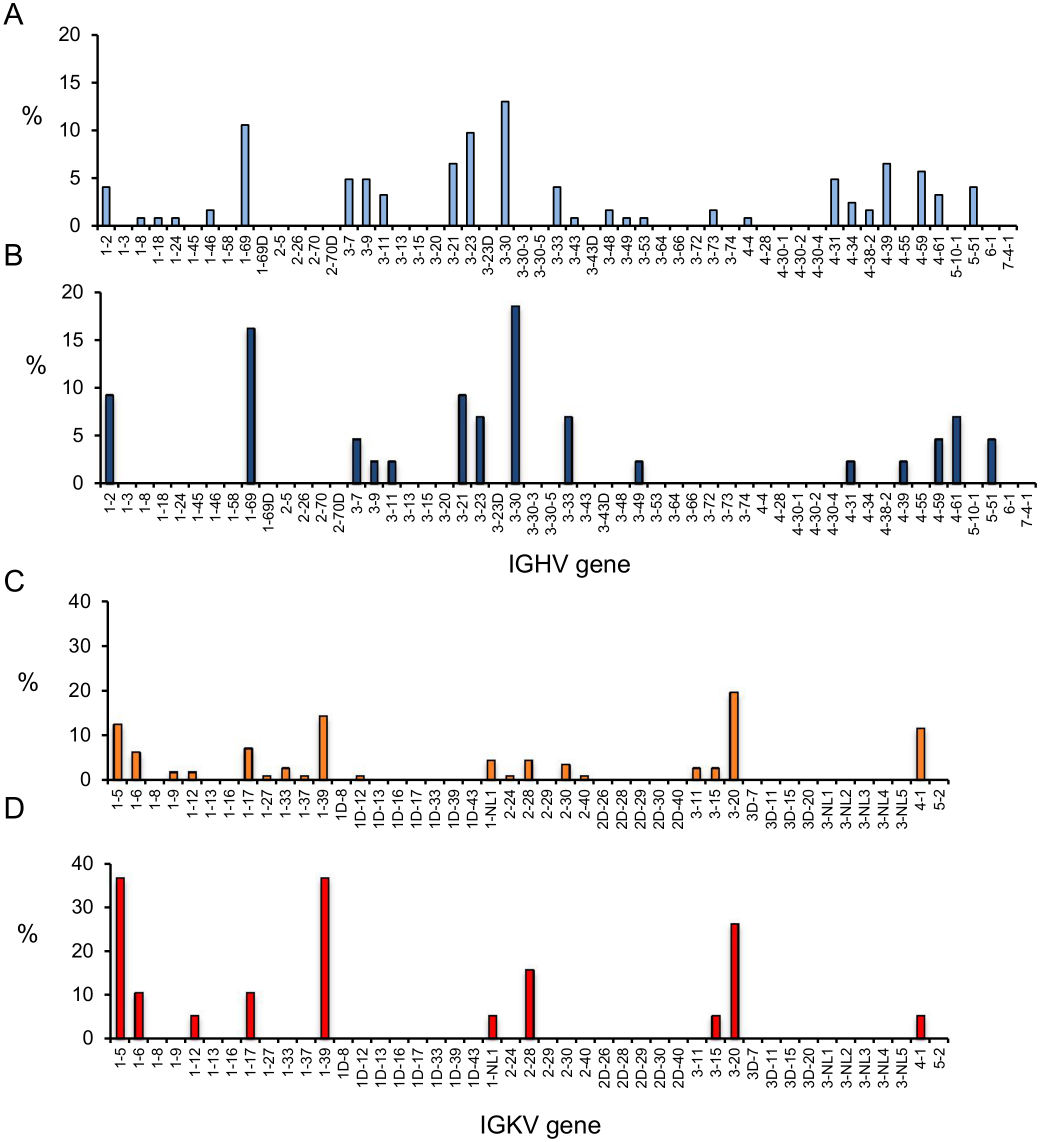
IGHV and IGKV germ-line gene usage. The IGHV and IGKV germ-line V gene usage was determined using the IMGT/HighV-Quest program. **A**) Percentage of individual IGHV genes of influenza NP-specific BcRs (N = 123). **B**) Percentage of IGHV genes of influenza NP-specific B cells that bound to influenza virus NP (N = 43). **C**) Percentage of IGKV genes of influenza virus NP-specific BcRs (N = 112). **D**) Percentage of IGKV genes used by influenza virus NP-specific binding antibodies (N = 31).

The HC genes originated from 26 different IGHV germ-line genes. The most frequently identified IGHV genes were 3–30 and 1–69. The LCκ genes originated from 19 different IGKV germ-line genes, including 18 V genes from the proximal V-gene cluster and a sequence that was allocated to the distal group of IGKV genes. The most frequently identified IGKV germ lines were 3–20, 1–39, and 1–5. A subset analysis of the sequences from functional antibodies showed similarly broad usage of the IGHV and IGKV germ-line genes. The V-gene usage did not differ between the identified BcRs and the antibodies that were functional in the ELISA (two-sided Wilcoxon rank tests; Fig 3).

### Clonally related memory B-cells

It was previously shown that influenza boost vaccination induces the pauciclonal expansion of B cells to plasma cells via intraclonal diversification through accumulated somatic mutations [5]. In a pilot experiment, we have seen that influenza vaccination increases the frequency of influenza NP-specific memory B cells in the peripheral blood in half of the subjects (data not shown). To examine if vaccination diversified the pool of influenza-specific memory B cells, we determined the number and size of the B-cell clones in the samples obtained after immunization and without prior vaccination, respectively. Cells that had HC sequences containing the same V and J genes and alleles, matching CDR3 lengths, and at least 70% V-gene sequence homology were regarded as potential clonal lineages. According to those criteria, 17% of the 123 HC sequences were from clonal lineages with two or more members. In the samples obtained after vaccination, four groups with 11 members of potential clonal lineages were identified. In the samples obtained without prior immunization, five groups with 10 members of potential clonal lineages were identified. The nucleotide sequences of the group members differed by 4.6–21.5% (Table 2).

**Table 2:** Genetic characteristics of potential clonal relatives.

Sample series^1^	V gene	J gene	CDR3 length (No. of amino acids)	Group size	Nucleotide sequence identity (%)
D1_s1	3–9*01	6*02	16	2	92.6
D1_s2	1–69*01	4*02	13	2	81.1
	3–30*03	4*02	15	2	86.6
	3–33*01	4*02	11	2	83.8
	4–39*01	4*02	15	2	78.5
D3_s2	1–2*02	4*02	12	4	83.8–92.3
	3–9*01	4*02	15	2	92.0
	3–30*14	6*02	20	2	95.4
D4_s2	4–31*03/ 4–31*06^2^	4*02	12	3	83.9–89.4

^1^ Number of sequences without boost vaccination (D1_s1, D1_s2, D2_s1): 64; Number of sequences after vaccination (D2_s2, D3_s2, D4_s2): 59

^2^ The allele of one group member was not unambiguous

### Wide variation of the heavy chain CDR3 lengths

**Fig 4:**
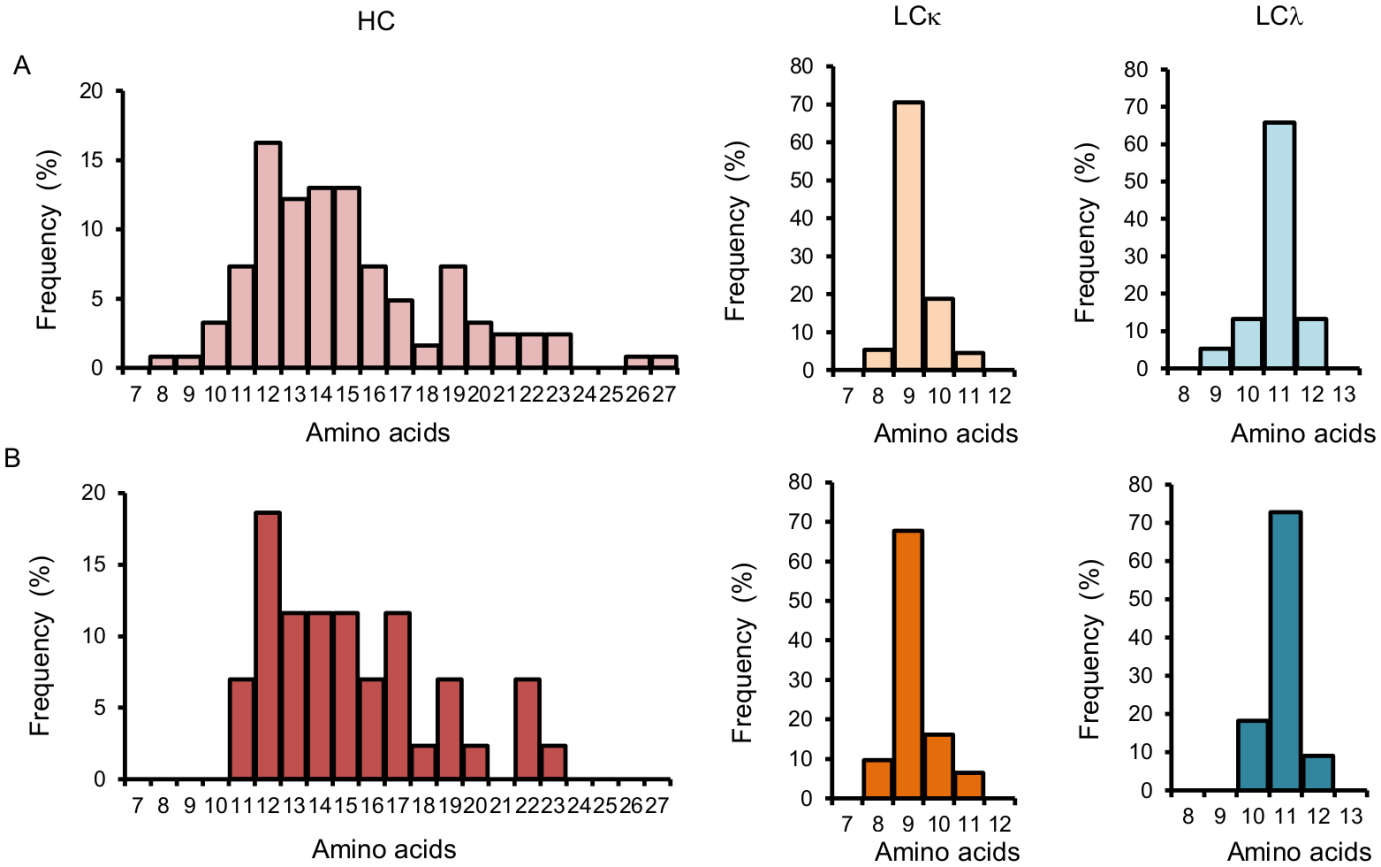
Number of amino acids in the CDR3 region. Percentages of BcR sequences of different lengths. **A**) Length distribution of heavy chain (HC), light chain (LC) κ and LCλ CDR3 sequences of all influenza NP-specific B cells. **B**) Length distribution in the CDR3 regions of gene sequence pairs that gave rise to functional antibodies.

The sequence of the variable region of the BcR of naïve B cells differs from the germ-line sequence at the junction of the V, D, and J genes in the HC CDR3 because of nucleotide additions and deletions during maturation from pro-B cells to naïve B cells. That gives naïve B cells a characteristic HC CDR3 length. The CDR3 length is maintained in most instances in affinity-matured memory B cells. The influenza NP-specific memory B cells showed a diverse repertoire of HC CDR3 lengths with a median length of 14 amino acids (range: 8–27). The LCκ had a median length of 9 amino acids (range: 5–11), and the CDR3 regions of the LCλ were slightly larger, with a median length of 11 amino acids (range: 9–12). The CDR3 length of the subsets of influenza NP-binding antibodies had a median of 15 (HC), 9 (LCκ), and 11 (LCλ) amino acids, respectively (Fig 4).

### High numbers of somatic mutations

**Fig 5:**
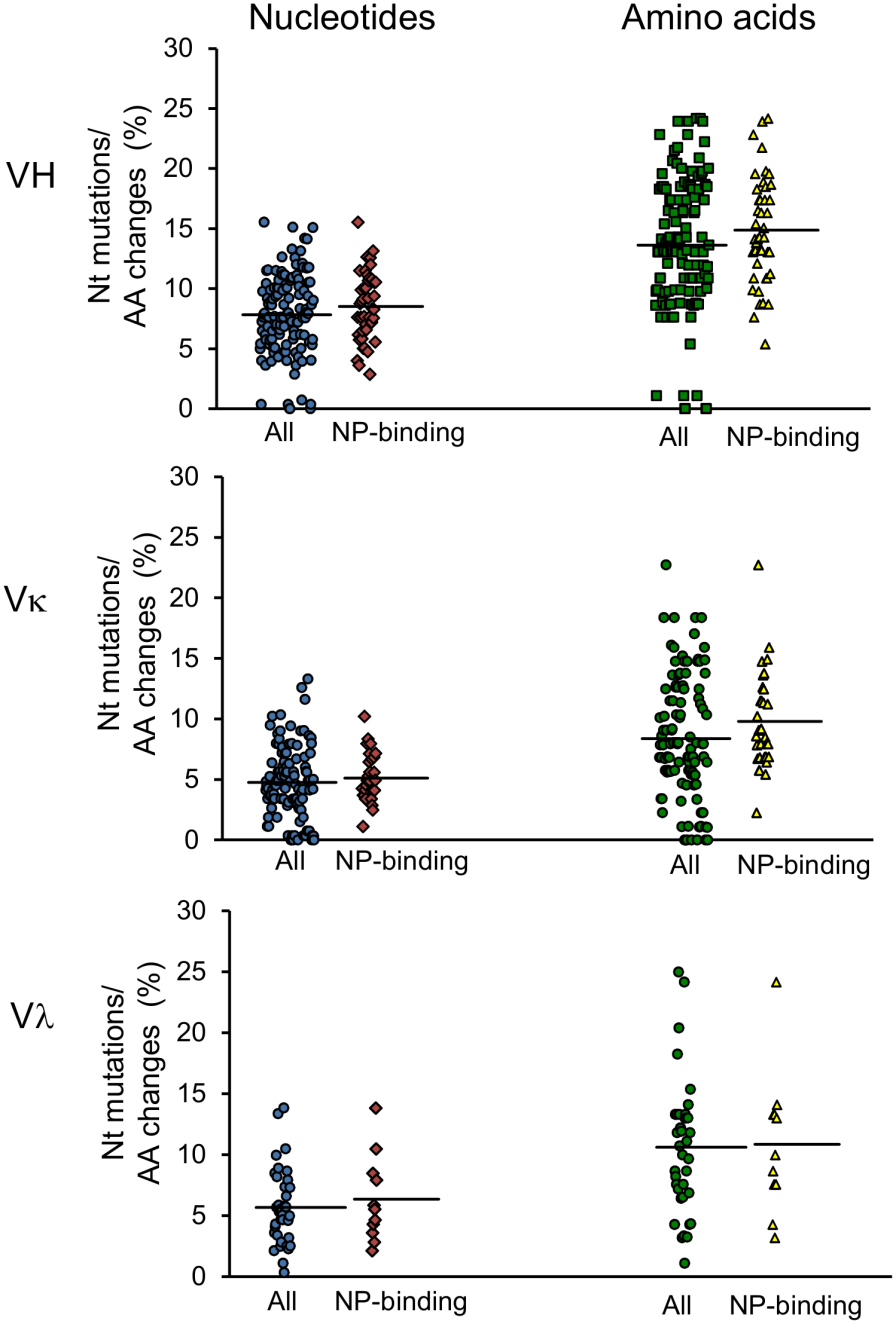
Nucleotide mutation rate and amino acid changes in the V genes. Percentages of point mutations and amino acid changes in the V gene compared with those in the germ-line sequences. Circles: all sequences of influenza NP-specific B cells (VH: N = 123, Vκ: N = 112, Vλ: N = 38); Triangles: subset of sequence pairs that gave rise to influenza NP-binding antibodies (VH: N = 43, Vκ: N = 31, Vλλ: N = 11).

Affinity maturation of B cells leads to variant progenies with point mutations in the variable regions of the HC and LC antibody genes. When compared with those in the germ-line alleles, the nucleotide sequences of the V gene of the HC in the influenza NP-specific cells showed a mean nucleotide sequence divergence of 7.8% and a mean amino acid sequence divergence of 13.6% (maximum 24.2%). The BcR analysis of the subset of influenza NP-binding antibodies gave similar values (8.6% nucleotide divergence and 14.9% amino acid divergence). The nucleotide sequences of the V gene of LCκ had an average of 4.7% nucleotide divergence, corresponding to 8.4% (maximum 22.7%) amino acid divergence (subset: 5% nucleotide divergence, 9.8% amino acid divergence). The LCλ exhibited a mean nucleotide divergence of 5.7%, corresponding to 10.6% (maximum 25%) amino acid divergence (subset: 6% nucleotide divergence and 10.8% amino acid divergence; Fig 5).

### High frequency of somatic insertions and deletions

In addition to the point mutations, we found insertions and deletions in 10 (8.1%) of the HC sequences seven of which belonging to functional influenza NP-specific antibodies. Four of the sequences (D3s2NP11, 14, 19, 38) came from a cluster of clonally related cells. The insertions and deletions were found in BcRs that used seven different V genes. The insertions were located in the CDR1, CDR2, or FR3 region, and the deletions were located in the CDR1 and FR3 regions. No insertions or deletions were found in the FR1 and FR2 regions. The insertions comprised 6–24 nucleotides, and the deletions comprised 3–9 nucleotides (Table 3).

**Table 3:** Somatic insertions and deletions in influenza NP-specific memory B-cells,

Antibody heavy chain	IGHV gene	IGHJ gene	I/D^a^	CDR/FR^b^	Codon position in V gene	Nucleotides	Influenza NP-binding
D1s12ZNP09h_mab	IGHV4-61*08	IGHJ5*01	D	CDR1	31/34	AGCAGT	√
D1s1NP20h_mab	IGHV3-21*01	IGHJ6*02	I	CDR2	62	GAGACT	√
D2s1NP34h1	IGHV3-30*17	IGHJ4*02	I	CDR1	27	CGAGACTCG	-
D3s2NP11h1_mab	IGHV1-2*02	IGHJ4*02	I	CDR2	64	CCATACATTGAT	√
D3s2NP19h3_mab	IGHV1-2*02	IGHJ4*02	I	CDR2	64	CCATACATTGAT	√
D3s2NP38h1_mab	IGHV1-2*02	IGHJ4*02	I	CDR2	64	CCATACATTGAT	√
D3s2NP14h1_mab	IGHV1-2*02	IGHJ4*02	I	FR3	66	GGAGTCACT	√
D4s2NP19h1	IGHV3-11*03	IGHJ1*01	I	CDR1	30	CTCAGTGGGTACCACATGACCTGG	-
D4s2NP33h1_mab	IGHV4-31*06	IGHJ6*02	D	CDR1	30	ATC	√
D4s2NP50h1	IGHV5-51*01	IGHJ6*02	D	FR3	90	CAGTGGAGC	-

^**a**^ I: Insertion; D: deletion

^**b**^ CDR: Complementarity-determining region, FR: Framework region

## Discussion

Efforts to generate vaccines using empirical methods for many viruses such as HIV and hepatitis C virus have not been successful. For a more rational approach to vaccine development, it will be informative to understand the B cell response at the level of the single antigen-specific cell. Influenza virus infections occur frequently in humans, and the immune response to the influenza virus can serve as a model system to explore the fine structure of the antiviral B cell response in humans. The influenza virus hemagglutinin glycoprotein is the primary target of virus-neutralizing antibodies. The high mutation rate of the hemagglutinin could affect the antibody response: antigens that mutate more rapidly might activate the B cell system differently than antigens that mutate more slowly. The influenza NP is more conserved among different influenza strains, making it an attractive target to examine the human B cell diversity formed by a slowly mutating viral protein.

We found considerable diversity among the memory B cells that developed in humans in response to influenza NP. Between 19 and 51 different influenza NP-specific memory B cells were found in each donor individual, and each individual had a distinct repertoire of antigen-specific cells. The number of different BcRs found in the experiments provides a minimum estimate for the BcR diversity among the donor individuals. The real BcR diversity is higher, because only a fraction of the B cells in each individual were examined. The high BcR diversity within and among the four individuals shows the enormous flexibility of the human B cell response and that the memory B cell response against the structurally conserved influenza NP is as diverse as the plasmablast response against the rapidly mutating influenza hemagglutinin [5, 7]. The high inter-individual B cell diversity provides evidence that host factors are important for the B cell repertoire.

The influenza NP-specific BcRs were composed of numerous HC and LC germ-line V genes. Several genes, including IGHV 1–69, 3–23, and 3–30 and IGKV 1–39 and 3–20, were found more frequently than others. Thus, the contribution to BcRs appears to be unequal rather than equal among different V genes. In the IMGT antibody database, which contains approximately 10,000 human BcR gene variants, IGHV 1–69 is the second most abundant HC V gene, and IGHV 3–23 and 3–30 are the third and fourth most abundant. Similarly, IGKV 3–20 and 1–39 are the most abundant LCκ genes in the IMGT database [27]. Thus, the usage of V genes by influenza NP-specific B cells roughly reflects that of other B cells. We did not identify gene sequences of IGHV subgroups 2, 6, and 7 in our analysis. Those subgroups were either not involved in influenza NP-specific BCRs, not detected because of suboptimal primer binding, or were rare in our samples.

We identified more than 100 natural variants of influenza NP-specific memory B cells. Each individual tested used a distinct subset of those cells. Influenza vaccination leads to the expansion and diversification of clonally related plasma cells [5]. To determine if the influenza NP-specific memory B cells showed signs of clonal diversification, the frequencies of groups of BcR sequences with the same HC V and J genes, the same CDR3 length, and at least 70% sequence homology were counted. We found several small groups of clonal relatives both before and after boost immunization. The nucleotide sequences of the potential clonal relatives differed by up to 20% or more. That variation is similar to that among the nucleotide sequences of individual BcRs from the germ line and reflects a high degree of diversification during previous influenza infections or vaccinations. The genetic diversity of the potential clonal relatives in the samples taken after boost immunization was between 5 and 16%. Based on a somatic mutation rate of 10^−3^ per base per generation [28], many rounds of cell division were required to accumulate the observed mutations. Hence, we think that the sequences represent B cells that were present before the boost vaccination and not those generated during the 2 weeks between the vaccination and the sampling. The number of antibody sequences studied was small, however, and subtle mutations may have been filtered out by our exclusion criteria. Vaccine-induced diversification of the memory B cell pool could become visible with larger sample sizes and later observation time points.

The CDR3 of the HC contained between eight and 27 amino acids, which is similar to the CDR3s of influenza-specific antibodies from plasmablasts [7] and most HIV gp140-specific B cells [4]. Approximately 10% of the influenza NP-specific antibodies had CDR3s of 22 amino acids and longer. Particularly long CDR3s have previously been found among broadly neutralizing HIV-specific antibodies such as the antibodies PG9 and PG16 that bind to the V1/V2 region of the gp120 glycoprotein. These antibodies have a CDR3 of 30 amino acids. The long CDR3 enables the antibodies to penetrate the glycan shield that covers the viral glycoprotein to attach to the protein surface of the molecule **(**[29, 30]. A similar loop-insertion binding mechanism has been described for the cross-neutralizing influenza hemagglutinin-specific antibody C05 that has a CDR3 of 24 amino acids [31]. It will be interesting to test if the long CDR3s in antibodies against the influenza NP similarly have a specific function.

The variability of the CDR3 length of the LC was much smaller in accordance with the restricted use of the terminal deoxynucleotidyl transferase during HC rearrangement during the pro-B cell stage before LC recombination. Similar to what has been shown for unselected B cells, the LCλ chain was slightly longer than the LCκ chain [32].

The average number of mutations in the HC V gene was higher than that previously found among non-selected IgG+ memory B-cells: 21.0 (7.8%) of 270 nucleotide mutations in this study versus 18 nucleotide mutations in IgG+ memory B-cells. It was also slightly higher than that found among influenza-specific plasmablasts: 19.4 mutations in the influenza plasmablast study by Wrammert et al. or 6% in the influenza study by Moody et al. [5, 7, 33]. This was surprising because most of the antibodies from plasmablasts were directed against the viral hemagglutinin that is rapidly mutating. It indicates that the B cell response to the structurally conserved influenza NP is as diverse as the antibody response to the more variable hemagglutinin. In comparison, HIV gp140-specific memory B cells had slightly more mutations than the influenza NP-specific antibodies [4]. Extraordinarily high numbers of point mutations (40 to 100) were reported in several broadly neutralizing HIV-specific antibodies [15], indicating an intense history of BcR adaptation to a structurally changing HIV antigen that was not seen among the influenza NP-specific BcRs.

It was previously shown that the BcRs of 0.3–4.6% of germinal-center and memory B cells contain somatic insertions and deletions [34–36]. We found evidence suggesting that insertions and deletions accumulated in the influenza NP-specific memory B cells. Somatic insertions in antigen-specific B cells were previously reported in B cell hybridomas [37] and in several antibodies against HIV and influenza virus. For instance, the HIV-specific cross-reactive neutralizing antibody VRC03 had a seven amino acid insertion in FR3. The broadly neutralizing HIV gp120-specific antibodies PGT125–128 contained a two amino-acid insertion in the CDR2 region, and the antibody NIH45-46 had a four amino-acid insertion in CDR3 [9, 38–40] reviewed in [41]. A recent study examined the frequency of insertions and deletions in a large set of broadly neutralizing HIV-specific antibodies and showed that the frequency of insertions and deletions was increased relative to that among other antibodies. The study also showed that the frequency of insertions and deletions was elevated in B cells from individuals infected with HIV compared with B cells from control individuals [14]. In addition, the influenza hemagglutinin-specific antibody 2D1 in humans contains an insertion of three amino acids in the FR3 region [42]. The high percentage of insertions and deletions found among influenza NP-specific B cells suggests that repeated exposure to the influenza virus, which likely occurs in most individuals, favors the accumulation of insertions and deletions.

The cloning of the variable HC and LC sequences and the expression of those sequences in HEK293T cells gave rise to 43 genetically distinct influenza NP-specific monoclonal antibodies. Previous studies using single-cell BcR amplification of isolated B cells yielded 134 HIV glycoprotein-specific antibodies from memory B cells from chronically infected individuals [4] and 61 or 46 clonally distinct antibodies against influenza virus from plasmablasts of vaccinated individuals [5, 7]. While the techniques used in this study and in the previous studies for obtaining antibody sequences differ in certain points, the number of antibodies obtained in each case was significantly higher than that achieved using conventional B cell transformation and hybridoma techniques. The direct antibody cloning methods allow the cloning of large and diverse sets of recombinant human antibodies. We obtained influenza antibody sequences from individuals without prior boost vaccination, suggesting that the technique can be applied for the production of therapeutic monoclonal antibodies against viruses for which no vaccine exists. A potential candidate is the Ebola virus, for which antibodies were shown to be partially protective [43].

In conclusion, our study shows that a large number of different memory B cells in each of four individuals were specific to influenza NP, highlighting the enormous diversity of the B cells among the individuals. The presence of a large and diverse set of B cells specific to a single protein suggests that a broad repertoire is advantageous for immune protection. The generation of recombinant human monoclonal antibodies without prior vaccination can be used to generate therapeutic human antibodies from individuals that have recovered from infection with highly pathogenic viruses.
