# Correction: High Inter-Individual Diversity of Point Mutations, Insertions, and Deletions in Human Influenza Virus Nucleoprotein-Specific Memory B Cells

**DOI:** 10.1371/journal.pone.0135320

**Published:** 2015-08-05

**Authors:** Sven Reiche, Yamen Dwai, Bianca M. Bussmann, Susanne Horn, Michael Sieg, Christian Jassoy

The affiliation for the fifth author is incorrect. Michael Sieg is not affiliated with #2. He is only affiliated with #1 Institute of Virology, Faculty of Medicine, University of Leipzig, Leipzig, Germany.
